# Development of HIV Drug‐Resistance Mutations and Antiretroviral Efficacy Among Vietnamese Patients After Failure of 5‐Year First‐Line Therapy

**DOI:** 10.1002/jcla.25157

**Published:** 2025-02-05

**Authors:** Than Manh Hung, Le Van Nguyen Bang, Le Van Duyet

**Affiliations:** ^1^ Emergency Department National Hospital for Tropical Diseases Hanoi Vietnam; ^2^ Infectious Department University of Medicine and Pharmacy, Vietnam National University Hanoi Vietnam; ^3^ Luong the Vinh High School Hanoi Vietnam; ^4^ Micobiology and Moclecular Biology Department National Hospital for Tropical Diseases Hanoi Vietnam

**Keywords:** drug resistance, first‐line therapy, HIV, mutation, virulence failure

## Abstract

**Background:**

The emergence of drug‐resistant mutations in human immunodeficiency virus (HIV) over time presents a challenge to treatment. We describe the development of drug‐resistance mutations and ART efficacy reduction in Vietnamese patients with failure of first‐line ART during a 5‐year period.

**Methods:**

This is a 5‐year observational cohort study with HIV viral loads of patients evaluated annually for 5 years (2017–2022) at the hospitals in Vietnam. Patients with a viral load ≥ 1000 copies/mL were subjected to identifying mutations in reverse transcriptase, protease, and integrase to evaluate HIV resistance and the efficacy of ART.

**Results:**

After 5 years of monitoring the HIV load of 2932 patients on ART, 75 (2.56%) patients had concurrent virological failure at all 5 years. In 2017, only 2/75 HIV strains possessed Protease Inhibitor (PI) resistance mutations, while 75/75 HIV strains had both Nucleoside Reverse Transcriptase Inhibitors (NRTIs) and Non‐Nucleoside Reverse Transcriptase Inhibitors (NNRTIs) resistance mutations. Only four PI resistance variants were found, while 40 and 32 mutations were resistant to NRTIs and NNRTIs. After 5 years, the number of HIV PI resistance mutations had increased to 14, with 13 new mutations emerging. There were six novel mutations associated with resistance to NRTIs, NNRTIs, and the proportion of preexisting mutations increased from 1.3% to 13.3%. Furthermore, HIV sensitivity to ART decreased from 2.7% to 18.6%.

**Conclusion:**

After 5 years, HIV had increased resistance mutations to PIs, NRTIs, and NNRTIs, with PI resistance mutations increasing the most rapidly, and the decrease in HIV sensitivity to PIs was higher than that to NRTIs and NNRTIs.

## Graphical abstract

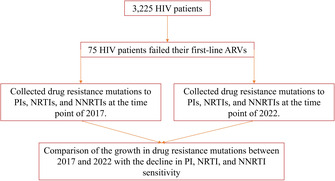

## 1 Introduction

HIV/AIDS continues to be the leading cause of disease burden and high mortality rates in developing countries, including Vietnam [1, 2]. HIV/AIDS remains a burden on both the health sector and the society in low‐resource countries, with high infection and mortality rates, as well as expensive and long‐term treatment expenses [3]. Approximately 79.3 million people worldwide are infected with HIV, of which 36.3 million have died [4]. As of 2020, 37.7 million and 28.2 million patients living with HIV are receiving antiretroviral therapy, respectively [4]. In Vietnam, approximately 250,000 people are living with HIV/AIDS in 2022, of which 180,000 are currently receiving ART (antiretroviral therapy) [5].

Currently, there is no specific treatment to completely eradicate HIV [6, 7, 8]. Therefore, antiretroviral (ARV) drugs to inhibit HIV replication, thereby prolonging the patient's life are the most effective solution [9, 10, 11]. According to the HIV/AIDS treatment and guidelines of the Ministry of Health of Vietnam [12], HIV‐infected adults or children over 10 years old who have not received ART or have no evidence of treatment failure are prescribed treatment with first‐line ART regimens, including: preferred regimens (tenofovir—TDF + lamivudine—3TC (or emtricitabine—FTC) + dolutegravir—DTG), alternative regimens (TDF + 3TC + *efavirenz*—EFV), and special regimens (TDF + 3TC (or FTC) + PI/r; TDF + 3TC (or FTC) + raltegravir—RAL; TAF = 3TC (or FTC) + DTG; abacavir—ABC + 3TC + DTG). If there is a contraindication for the preferred or alternative treatment, use only the alternative or special treatment regimen. In addition, this guideline recommends switching from a first‐line ART regimen to a TLD regimen (TDF 300 mg, 3TC 300 mg, and DTG) 50 mg if drug resistance develops. Patients who fail therapy with first‐line ART or second‐line ART regimens are allocated to second‐line ART regimens (AZT + 3TC + LPV/r/DRV/r/DTG) or third‐line ART regimens (danuravir—DRV/r + 2 NRTIs ± DTG), respectively. Treatment with HIV/AIDS must be maintained for life [7]. Over a long period of time, patients face many challenges, such as treatment compliance, drug side effects, and most notably viral drug resistance [13, 14]. HIV has a very high resistance to first‐line ART drugs. A large number of patients had to switch to second‐line ART, while others are now on third‐line ART [15]. In Vietnam, 7.2% of patients demonstrated ART therapy failed within 12 months of treatment, necessitating a switch to a more suitable regimen to prevent viral flare‐up and new infections [16]. As a result, expanding the use of ARTs necessitates rigorous monitoring of drug resistance and the appropriate selection of second‐ or third‐line treatment in the event of failure of first‐line treatment. In addition, effective prevention of new HIV infection will be the most important step toward resolving medical and budgetary issues [17, 18].

There have been several reports on the development of HIV drug resistance in Vietnamese patients who have failed first‐ and second‐line ART [19, 20, 21, 22, 23, 24, 25]. These studies were conducted with a limited sample size and short follow‐up, as well as there has been a significant time gap. Therefore, the objective of this study was to describe the treatment failure rate of HIV patients with a large sample size, multiple hospitals, an update and over a long period of time, as well as to assess the increase in drug‐resistance mutations and the decline in ART efficacy that are currently used in HIV treatment.

## 2 Materials and Methods

### 2.1 Study Setting, Design, and Sampling

We conducted a prospective, longitudinal study to investigate and monitor drug resistance in patients with HIV receiving ART. Criteria for patient selection included being a Vietnamese citizen, 16 years old, having a positive HIV test, receiving ART treatment for ≥ 6 months prior to Oct 2017, consenting to participate in the study, and signing an informed consent form. All patients who did not comply with treatment were excluded from the study.

HIV viral load measurements were performed every 1 year for 5 years on 3225 patients. Demographic characteristics, epidemiology, patient history, and previous treatment regimens were collected. During the 5‐year follow‐up period, 293 patients were excluded from the study due to loss of follow‐up, death, and transfer to another clinic. Over a 5‐year period, only 2932 patients maintained comprehensive viral load monitoring, with 115 patients had virological failure at ≥ 1 timepoint and 75 experiencing virological failure at all five timepoints (2017, 2018, 2019, 2020, 2021, and 2022). Therefore, we selected all of 75 patients to compare the development of HIV drug‐resistance mutations as well as the decline in ART sensitivity after 5 years since the patient's diagnosis of first‐line ART resistance.

Blood samples were collected in tubes containing EDTA anticoagulant, and plasma was separated and stored at −80°C. The plasma sample was delivered to the central laboratory of NHTD for viral load measurement, genotyping, and drug‐resistance identification. Protease (PR), reverse transcriptase (RT), and integrase (IN) gene sequencing were performed to identify drug‐resistance mutations in patients with HIV RNA ≥ 1.000 copies/mL (virological failure). We did not intervene in medical facilities' ART treatment processes but rather provided clinicians with information about the viral load and drug‐resistance status of patients with HIV. Therefore, all activities related to the diagnosis and treatment of patients with HIV were carried out in accordance with the guidelines of the Ministry of Health of Vietnam and the actual situation at medical facilities.

### 2.2 Data Collection

Medical collection forms were used to collect information on HIV drug resistance test results as of 2017 and 2022, as well as patient demographics, epidemiology, and ART treatment. We were licensed to collect all information of 75 HIV‐infected patients being monitored at the hospitals in Vietnam and collected data on an information collection form specifically designed for this study. The full names of all HIV‐infected patients were encrypted in accordance with the regulations of the Vietnamese Ministry of Health.

### 2.3 Laboratory Tests

The viral load of HIV samples was measured using COBAS/TaqMan HIV Test v2.0 (Roche Diagnostics, Rotkreuz, Switzerland) commercial kit. Patients with virological failure (confirmed when viral load is ≥ 1000 copies/mL plasma) were sequenced using Sanger technique to detect drug‐resistance mutations.

Drug‐resistance‐related mutations in the protease (PR), reverse transcriptase (RT), and integrase (IN) genes as well as genotypes of 75 HIV samples were analyzed in NHTD central molecular diagnostic laboratory in compliance with the SOP developed by the NHTD. Plasma samples collected from patients were used to extract total RNA using Qiagen's kit (QIAamp Viral RNA Mini Kit, Cat No. 52904, Germany), and then the RNA was directly used for PCR reactions to amplify IN, PR, and RT genes. IN, PR, and RT genes were amplified in two steps using primers and protocols as described in the Appendix A: RT‐PCR and nested PCR. Following amplification, IN, PR, and RT PCR products were sequenced on an Applied Biosysem 3500 Genetic Analyzer (Applied Biosystems, Carlsbad, CA, USA) with a BigDye Terminator v1.1 Cycle Sequencing Kit and a BigDye XTerminator Purification Kit (Applied Biosystems).

IN, PR, and RT sequences of the HIV samples were analyzed and edited using MEGA software (version 11.0.10) [26]. FASTA files containing sequences were subsequently uploaded to the HIVdb program provided by the Stanford HIV Database [27, 28]. The IN, PR, and RT gene sequences have been submitted to NCBI Genbank with the submission ID 2913573, with a release date of July 18, 2025. The following mutations and levels of ART resistance were assigned as follows: “Susceptible” indicates no evidence of decreased ART susceptibility. “Potential low‐level resistance” refers to the presence of mutations that indicate previous ARV exposure or mutations that have been associated with drug resistance only when they occur in the presence of other mutations. “Low‐level resistance” refers to mutations that confer decreased ARV susceptibility in vitro or mutations that result in a poor virological response to ARV treatment in a patient. “Intermediate resistance” denotes a high probability of decreased drug activity, whereas “high‐level resistance” denotes an expected level of resistance similar to that seen in viruses with the highest levels of in vitro drug resistance, or clinically, that people living with HIV (PLHIV) infected with these viruses had little to no virological response to ARV treatment [27, 28].

### 2.4 Data Analysis

Statistical analyses were performed using SPSS version 29.0.1 statistical software [29]. We calculated the mean and IQR of patient years old as the difference between the upper and lower quartiles and performed simple and multivariate logistic regression analysis to determine the percentages of demographic variables, epidemiology, and treatment of patients. Separate analysis and comparison of the increase in mutation rate and ART resistance level over time were performed using GraphPad Prism software (GraphPad Software Inc., San Diego, CA, USA).

## 3 Results

Viral load monitoring of 2932 patients revealed virological failure in 115 patients during the 5‐year follow‐up. In 2017, 2018, 2019, 2020, 2021, and 2022, there were 102, 88, 91, 85, 57, and 82 HIV patients with virological failure, respectively (Table 1). TLE, TLD, and several combination regimens such as TDF/3TC/LPV, ABC/LPV/3TC, AZT/3TC/LPV, ABC/3TC/DTG are the main ART regimens used, in which 35.8% of patients were received ART therapy with DTG. Among 115 HIV patients with virological failure at ≥ 1 timepoint, there were 75 patients with concurrent virological failure at all five timepoints (2017, 2018, 2019, 2020, 2021, and 2022), these patients were selected to compare drug‐resistance mutation development of HIV (Table 1).

**TABLE 1:** Viral load and ART regimen status of HIV‐infected patients during the 5‐year study period (*n* = 2932).

Number of patients	Timepoint/viral load/ART regimen					
	2017	2018	2019	2020	2021	2022
2817						
2	TLE	TLD	TLD	TLD	TLD	TLD
1	TLE	TLE	TLE	TLD	TLD	TLD
1	TLE	TLD	TLE	TLE	TLD	TLD
2	TLE	TLE	TLE	TLE	TLE	TLD
1	TLE	TLE	TLE	TLD	TLD	TLD
2	TLE	TLE	TLE	TLD	TLD	TLD
2	TDF/3TC/LPV	TDF/3TC/LPV	TDF/3TC/LPV	TLD	TLD	TLD
1	ABC/LPV/3TC	ABC/LPV/3TC	TLD	TLD	TLD	TLD
1	TLE	TLD	TLD	TLD	TLD	TLD
1	TLE	TLE	TLE	TLD	TLD	TLD
2	TDF/3TC/LPV	TDF/3TC/LPV	TLD	TLD	TLD	TLD
1	TLE	TLE	TLD	TLD	TLD	TLD
1	TDF/3TC/LPV	TDF/3TC/LPV	TLD	TLD	TLD	TLD
3	AZT/3TC/LPV	AZT/3TC/LPV	AZT/3TC/LPV	AZT/3TC/LPV	AZT/3TC/LPV	AZT/3TC/LPV
1	TLE	TLE	TLE	TLD	TLD	TLD
1	ABC/3TC/EFV	ABC/3TC/EFV	ABC/3TC/EFV	ABC/3TC/DTG	ABC/3TC/DTG	ABC/3TC/DTG
1	ABC/3TC/EFV	ABC/3TC/EFV	ABC/3TC/LPV	ABC/3TC/DTG	ABC/3TC/DTG	ABC/3TC/DTG
1	AZT/3TC/LPV	AZT/3TC/LPV	AZT/3TC/LPV	AZT/3TC/LPV	TLD	TLD
1	TLE	TDF/3TC/LPV	TDF/3TC/LPV	TDF/3TC/LPV	TDF/3TC/LPV	TDF/3TC/LPV
1	TDF/3TC/LPV	TDF/3TC/LPV	TDF/3TC/LPV	TDF/3TC/LPV	TDF/3TC/LPV	TDF/3TC/LPV
1	TLE	TDF/3TC/LPV	TDF/3TC/LPV	TDF/3TC/LPV	TDF/3TC/LPV	TDF/3TC/LPV
1	TLE	TLE	TLE	TLE	TLE	TLE
1	TLE	TLE	TLE	TLD	TLD	TLD
2	TLE	AZT/3TC/LPV	AZT/3TC/LPV	AZT/3TC/LPV	AZT/3TC/LPV	AZT/3TC/LPV
1	TLE	TLE	TLE	TLD	TLD	TLD
1	TDF/3TC/LPV	TDF/3TC/LPV	TDF/3TC/LPV	TDF/3TC/LPV	TDF/3TC/LPV	TDF/3TC/LPV
1	TDF/3TC/LPV	TLD	TLD	TLD	TLD	TLD
2	TDF/3TC/LPV	TLD	TLD	TLD	TLD	TLD
1	TDF/3TC/LPV	TLD	TLD	TLD	TLD	TLD
1	TLE	TLE	TLE	TLD	TLD	TLD
1	AZT/3TC/LVP	AZT/3TC/LPV	AZT/3TC/LPV	AZT/3TC/LPV	AZT/3TC/LPV	AZT/3TC/LPV
11	TLE	TLE	TLE	TLD	TLD	TLD
5	TDF/3TC/LPV	TDF/3TC/LPV	TDF/3TC/LPV	TDF/3TC/LPV	TDF/3TC/LPV	TDF/3TC/LPV
6	TLE	TLE	AZT/3TC/LPV	AZT/3TC/LPV	AZT/3TC/LPV	AZT/3TC/LPV
5	TDF/3TC/LPV	TDF/3TC/LPV	TDF/3TC/LPV	TDF/3TC/LPV	TDF/3TC/LPV	TDF/3TC/LPV
15	TLE	TLD	TLD	TLD	TLD	TLD
6	TLE	TLE	AZT/3TC/LPV	AZT/3TC/LPV	AZT/3TC/LPV	AZT/3TC/LPV
17	TLE	TLE	TDF/3TC/LPV	TDF/3TC/LPV	TDF/3TC/LPV	TDF/3TC/LPV
10	AZT/3TC/LPV	AZT/3TC/LPV	AZT/3TC/LPV	AZT/3TC/LPV	AZT/3TC/LPV	AZT/3TC/LPV
2932	☐ = 2826 ☐ = 4 ☐ = 102	☐ = 2829 ☐ = 15 ☐ = 88	☐ = 2832 ☐ = 9 ☐ = 91	☐ = 2835 ☐ = 12 ☐ = 85	☐ = 2840 ☐ = 35 ☐ = 57	☐ = 2840 ☐ = 10 ☐ = 82

☐	< 20 copies/mL	☐	≥ 20 – < 1000 copies/mL	☐	≥ 1000 copies/mL

*Note:* TLE: (TDF/3TC/EFV); TLD: (TDF/3TC/DTG); TDF: tenofovir disoproxil fumarate; 3TC: lamivudine; LPV: lopinavir; AZT: zidovudine; DTG: dolutegravir; EFV: *efavirenz*; ABC: abacavir.

### 3.1 Demographic and Baseline Characteristics

Among the 75 patients included in the study, the mean age was 43 years, the youngest was 21 years and the oldest was 73 years. There were 62.7% of men and 37.3% of women. The most common mode of transmission was sexual contact (68%), followed by mother‐to‐infant contact (15%), blood, needles (1%), and other causes (16%). In total, 53.3%, 33.4%, and 13.3% of patients lived in urban, rural, and mountainous areas, respectively (Table 2). The duration of HIV infection between 5 and10 years was 66.7%, 26.7% (11–15 years), and 6.6% (16–> 20 years). The most common subtype was CRF01_AE (72%), followed by subtype A (24%) and subtype B (4%) (Table 2).

**TABLE 2:** Demographic and baseline characteristics of the patients (*n* = 75).

Characteristics	*n*	%
Age (years old – 2017)		
Median (Min–Max)	43 (21–73)	
Gender		
Male	47	62.7
Female	28	37.3
Transmission route		
Sexual contact	51	68.0
Mother to infant	11	14.7
Blood, needles	1	1.3
Others	12	16.0
Geographic distribution		
Mountain	10	13.3
Rural	25	33.4
Urban	40	53.3
HIV infection time (years)		
5–10	50	66.7
11–15	20	26.7
16–> 20	5	6.6
HIV subtype		
A	18	24.0
B	3	4.0
CRF01_AE	54	72.0

### 3.2 HIV Drug Resistance Mutations to PIs, NRTIs, and NNRTIs Between 2017 and 2022

The IN, PR, and RT genes of 75 HIV samples were successfully nucleotide sequenced; nevertheless, no alterations were found in the IN gene of all 75 HIV samples. In 2017, only two patients infected with HIV strains exhibited mutations resistant to protease inhibitors, including L10F, M46I, M46L, I54V, and V82A, which accounted for 1.3% (Table 3). However, by 2022, the number of HIV strains with protease resistance mutations had risen to 14, with alterations recognized in 2017 such as L10F, M46I, M46L, I54V, and V82A increasing by 2.7%, 10.7%, 2.7%, 4.0%, and 2.7%, respectively. In addition, the following numbers of novel mutations were recorded: L10V (1.3%), L24I (1.3%), I47A (2.7%), F53L (2.7%), Q58E (2.7%), G73S (1.3%), T74S (1.3%), V82F (1.3%), V82C (1.3%), I84V (1.3%), N88T (1.3%), L89V (5.3%), and L90M (4.0%) (Table 3).

**TABLE 3:** Protease‐resistance mutations after first‐line antiretroviral therapy and 5 years after treatment.

Mutation	2017 (*n* = 2/75)	2022 (*n* = 14/75)	Number of mutations increased
L10F	1 (1.3)	3 (4.0)	2 (2.7)
L10V	0 (0.0)	1 (1.3)	1 (1.3)
L24I	0 (0.0)	1 (1.3)	1 (1.3)
M46I	1 (1.3)	6 (8.0)	5 (10.7)
M46L	1 (1.3)	3 (4.0)	2 (2.7)
I47A	0 (0.0)	2 (2.7)	2 (2.7)
F53L	0 (0.0)	2 (2.7)	2 (2.7)
I54V	1 (1.3)	4 (5.3)	3 (4.0)
Q58E	0 (0.0)	2 (2.7)	2 (2.7)
G73S	0 (0.0)	1 (1.3)	1 (1.3)
T74S	0 (0.0)	1 (1.3)	1 (1.3)
V82F	0 (0.0)	1 (1.3)	1 (1.3)
V82C	0 (0.0)	1 (1.3)	1 (1.3)
V82A	1 (1.3)	3 (4.0)	2 (2.7)
I84V	0 (0.0)	1 (1.3)	1 (1.3)
N88T	0 (0.0)	1 (1.3)	1 (1.3)
L89V	0 (0.0)	4 (5.3)	4 (5.3)
L90M	0 (0.0)	3 (4.0)	3 (4.0)

As of 2017, all 75 patients were infected with HIV strains that are resistant to NRTIs. High‐frequency mutations included M41L (28.0%), K65R (29.3%), D67N (24.0%), K70R (25.3%), V75M (49.3%), M184V (81.3%), L210W (14.7%), T215F (28.0%), and K219E (24.0%) (Table 4). In addition, 31 additional mutations were identified, with incidence rates ranging from 1.3% to 912.0%. By 2022, M41L, and K65R mutations had not increased, but D67N (up 8.0%), K70R (up 1.3%), V75M (up 13.3%), M184V (up 8.0%), L210W (up 4.0%), T215F (up 6.7%), and K219E (up 9.3%) (Table 4). Several additional variations had large increases, including L74I (from 12.0% to 20.0%), F77L (5.3% to 10.7%), S68G (9.3% to 13.3%), Y115F (4.0% to 9.3%), and K70E (6.7% to 12.0%). Several novel variants emerged in 2022, including T69G (4.0%), T69S (1.3%), D75I (1.3%), K110E (1.3%), G190S (1.3%), and L210Y (1.3%) (Table 4).

**TABLE 4:** Resistance mutations of NRTIs during first‐line antiretroviral therapy and after 5 years.

Mutation	2017 (*n* = 75/75)	2022 (*n* = 75/75)	Number of mutations increased
M41L	21 (28.0)	21 (28.0)	0 (0.0)
E44D	4 (5.3)	4 (5.3)	0 (0.0)
A62V	3 (4.0)	3 (4.0)	0 (0.0)
K65R	22 (29.3)	22 (29.3)	0 (0.0)
K65N	1 (1.3)	1 (1.3)	0 (0.0)
K65E	1 (1.3)	1 (1.3)	0 (0.0)
D67N	18 (24.0)	24 (32.0)	6 (8.0)
D67S	6 (8.0	6 (8.0	0 (0.0)
D67G	6 (8.0	6 (8.0	0 (0.0)
D67H	1 (1.3)	1 (1.3)	0 (0.0)
D67T	2 (2.7)	4 (5.3)	2 (2.7)
S68G	7 (9.3)	10 (13.3)	3 (4.0)
S68N	3 (4.0)	5 (6.7)	2 (2.7)
S68R	1 (1.3)	1 (1.3)	0 (0.0)
T69D	5 (6.7)	5 (6.7)	0 (0.0)
T69G	0 (0.0)	3 (4.0)	3 (4.0)
T69S	0 (0.0)	1 (1.3)	1 (1.3)
K70E	5 (6.7)	9 (12.0)	4 (5.3)
K70G	4 (5.3)	5 (6.7)	1 (1.3)
K70T	3 (4.0)	4 (5.3)	1 (1.3)
K70R	19 (25.3)	20 (26.7)	1 (1.3)
K70N	2 (2.7)	3 (4.0)	1 (1.3)
K70Q	1 (1.3)	1 (1.3)	0 (0.0)
L74I	9 (12.0)	15 (20.0)	6 (8.0
L74V	7 (9.3)	7 (9.3)	0 (0.0)
V75M	37 (49.3)	47 (62.7)	10 (13.3)
V75I	5 (6.7)	6 (8.0)	1 (1.3)
D75I	0 (0.0)	1 (1.3)	1 (1.3)
F77L	4 (5.3)	8 (10.7)	4 (5.3)
K110E	0 (0.0)	1 (1.3)	1 (1.3)
Y115F	3 (4.0)	7 (9.3)	4 (5.3)
F116Y	3 (4.0)	4 (5.3)	1 (1.3)
Q151M	4 (5.3)	6 (8.0	2 (2.7)
M184V	61 (81.3)	67 (89.3)	6 (8.0
M184I	8 (10.7)	11 (14.7)	3 (4.0)
G190S	0 (0.0)	1 (1.3)	1 (1.3)
L210W	11 (14.7)	14 (18.7)	3 (4.0)
L210Y	0 (0.0)	1 (1.3)	1 (1.3)
T215F	21 (28.0)	26 (34.7)	5 (6.7)
T215Y	6 (8.0	11 (14.7)	5 (6.7)
T215S	1 (1.3)	3 (4.0)	2 (2.7)
T215I	1 (1.3)	3 (4.0)	2 (2.7)
K219Q	6 (8.0	7 (9.3)	1 (1.3)
K219E	18 (24.0)	25 (33.3)	7 (9.3)
K219N	3 (4.0)	5 (6.7)	2 (2.7)
K219R	2 (2.7)	5 (6.7)	2 (2.7)

In 2017, all 75 HIV strains with drug‐resistant mutations were identified, including high‐frequency variants such as A98G (18.7%), K101E (24.0%), K103N (38.7%), V108I (25.3%), Y181C (37.3%), G190A (30.7%), H221Y (13.3%), P225H (13.3%), and L101I (12.0%) (Table 5). In addition, 23 other mutations appeared with rates ranging from 1.3% to 9.3% (Table 5). In addition, 23 additional mutations appeared, with rates ranging from 1.3% to 9.3% (Table 5). By 2022, previously occurring mutations such as A98G (4.0%), K101E (2.7%), K101N (4.0%), V106I (6.7%), V108I (8.0%), Y181C (13.3%), G190A (13.3%), and H221Y (9.3%) had all increased (Table 5). Other mutations also increased in frequency (1.3%–5.3%). In addition, some novel variants were observed in 2022, including: K101H (4.0%), K101I (1.3%), K103H (1.3%), K103S (1.3%), E138Q (1.3%), and Y188F (1.3%) (Table 5).

**TABLE 5:** Resistance mutations of NNRTIs during first‐line antiretroviral therapy and after 5 years.

Mutation	2017 (*n* = 75/75)	2022 (*n* = 75/75)	Number of mutations increased
V10I	1 (1.3)	1 (1.3)	0 (0.0)
A98G	14 (18.7)	17 (22.7)	3 (4.0)
L100I	9 (12.0)	10 (13.3)	1 (1.3)
L100V	1 (1.3)	1 (1.3)	0 (0.0)
K101P	2 (2.7)	3 (4.0)	1 (1.3)
K101E	18 (24.0)	20 (26.7)	2 (2.7)
K101F	1 (1.3)	1 (1.3)	0 (0.0)
K101F	1 (1.3)	2 (2.7)	1 (1.3)
K101H	0 (0.0)	3 (4.0)	3 (4.0)
K101I	0 (0.0)	1 (1.3)	1 (1.3)
K103N	29 (38.7)	32 (42.7)	3 (4.0)
K103H	0 (0.0)	1 (1.3)	1 (1.3)
K103S	0 (0.0)	1 (1.3)	1 (1.3)
V106I	7 (9.3)	12 (16.0)	5 (6.7)
V106M	4 (5.3)	7 (9.3)	3 (4.0)
V108I	19 (25.3)	25 (33.3)	6 (8.0)
E138Q	0 (0.0)	1 (1.3)	1 (1.3)
V179D	7 (9.3)	9 (12.0)	2 (2.7)
V179E	1 (1.3)	1 (1.3)	0 (0.0)
V179F	1 (1.3)	1 (1.3)	0 (0.0)
V179T	1 (1.3)	1 (1.3)	0 (0.0)
Y181I	2 (2.7)	3 (4.0)	1 (1.3)
Y181G	1 (1.3)	1 (1.3)	0 (0.0)
Y181C	28 (37.3)	38 (50.7)	10 (13.3)
Y188L	7 (9.3)	11 (14.7)	4 (5.3)
Y188C	5 (6.7)	5 (6.7)	0 (0.0)
Y188F	0 (0.0)	1 (1.3)	1 (1.3)
G190A	23 (30.7)	33 (44.0)	10 (13.3)
G190Q	2 (2.7)	2 (2.7)	0 (0.0)
G190T	1 (1.3)	2 (2.7)	1 (1.3)
G190S	3 (4.0)	4 (5.3)	1 (1.3)
H221Y	10 (13.3)	17 (22.7)	7 (9.3)
P225H	10 (13.3)	12 (16.0)	2 (2.7)
F227C	1 (1.3)	2 (2.7)	1 (1.3)
G227L	1 (1.3)	1 (1.3)	0 (0.0)
F227L	1 (1.3)	1 (1.3)	0 (0.0)
M230L	7 (9.3)	7 (9.3)	0 (0.0)
K238T	5 (6.7)	5 (6.7)	0 (0.0)

### 3.3 Reduction of HIV Susceptibility to PIs, NRTIs, and NNRTIs Between 2017 and 2022

When HIV mutations associated with drug resistance were compared to ART regimens used in treatment, it was found that none of the seven regimens employed in this study were associated with resistance to integrase inhibitors (INIs). Meanwhile, the ABC/3TC/DTG regimen did not result in any resistance mutations to PIs, NRTIs, or NNRTIs. The two regimens ABC/3TC/EFV and TLE did not result in PI‐resistant mutations; however, they produced NRTI and NNRTI‐resistant mutations (Table S1). The four regimens: ABC/3TC/LPV, AZT/3TC/LPV, TDF/3TC/LPV, and TLD all result in resistance mutations to PIs, NRTIs, and NNRTIs. Although the ABC/3TC/LPV regimen produced fewer mutations resistant to NRTIs and NNRTIs than AZT/3TC/LPV, TDF/3TC/LPV, and TLD, the mutation rate and number of uses were much higher. Notably, even though the LDT regimen does not contain anti‐PIs, it produced PIs‐resistant mutations in the same way as LPVr‐containing regimens. The ABC/3TC/EFV regimen had the fewest mutations, followed by ABC/3TC/LPV, and AZT/3TC/LPV. TDF/3TC/LPV; TLD; and TLE (Table S1).

In 2017, the sensitivity rates of HIV strains to PIs were still high (ranging from 97.3% to 100.0%); however, by 2022, HIV strains' sensitivity to PIs had dropped significantly, with ATV/r decreasing by 17.3%, DRV/r decreasing by 6.7%, and LPV/r decreasing by 18.6% (Table 6). In 2017, only AZT and TDF were sensitive at 44.0% and 17.3%, respectively. However, by 2022, this percentage had dropped to just 28.0% and 10.7%. In 2017, the sensitivity rate of HIV to NNRTI was still fairly low (varying from 2.7% to 4.0%), and by 2022, it will be only 1.3% (Table 6). In contrast, the rate of HIV strains resistant to PIs, NRTIs, and NNRTIs all increased rapidly, with HIV high resistance to both NRTIs and NNRTIs being the highest, particularly to AZT (from 42.7% to 54.7%), ABC (from 64.0% to 73.3%), DOR (from 29.3% to 52.0%), EFV (from 86.7% to 98.7%), ETR (from 30.7% to 46.7%), and RPV (from 70.7% to 85.3%) (Table 6). HIV low and intermediate resistance to NRTIs and NNRTIs tended to reduce or rise insignificantly with time. Meanwhile, HIV resistant to PIs demonstrated enhanced resistance to all three medications (ATV/r, DRV/r, and LPV/r) (Table 6).

**TABLE 6:** Comparison of reduction of HIV susceptibility to ART drugs between 2017 and 2022.

Drugs	Antiretroviral susceptibility levels							
	Sensitive		Low resistance		Intermediate resistance		High resistance	
	2017	2022	2017	2022	2017	2022	2017	2022
ATV/r	73 (97.3)	60 (80.0)	0 (0.0)	5 (6.7)	1 (1.3)	5 (6.7)	1 (1.3)	5 (6.7)
DRV/r	75 (100.0)	70 (93.3)	0 (0.0)	4 (5.3)	0 (0.0)	1 (1.3)	0 (0.0)	0 (0.0)
LPV/r	73 (97.3)	59 (78.7)	0 (0.0)	9 (12.0)	1 (1.3)	0 (0.0)	1 (1.3)	7 (9.3)
ABC	0 (0.0)	0 (0.0)	10 (13.3)	4 (5.3)	17 (22.7)	16 (21.3)	48 (64.0)	55 (73.3)
AZT	33 (44.0)	21 (28.0)	6 (8.0)	10 (13.3)	4 (5.3)	3 (4.0)	32 (42.7)	41 (54.7)
FTC	1 (1.3)	1 (1.3)	2 (2.7)	0 (0.0)	3 (4.0)	2 (2.7)	69 (92.0)	72 (96.0)
3TC	1 (1.3)	1 (1.3)	2 (2.7)	0 (0.0)	3 (4.0)	2 (2.7)	69 (92.0)	72 (96.0)
TDF	13 (17.3)	8 (10.7)	16 (21.3)	18 (24.0)	29 (38.7)	27 (36.0)	17 (22.7)	22 (29.3)
DOR	2 (2.7)	0 (0.0)	14 (18.7)	5 (6.7)	37 (49.3)	31 (41.3)	22 (29.3)	39 (52.0)
EFV	0 (0.0)	0 (0.0)	1 (1.3)	0 (0.0)	9 (12.0)	1 (1.3)	65 (86.7)	74 (98.7)
ETR	3 (4.0)	1 (1.3)	14 (18.7)	5 (6.7)	35 (46.7)	33 (44.0)	23 (30.7)	35 (46.7)
NVP	0 (0.0)	0 (0.0)	0 (0.0)	0 (0.0)	0 (0.0)	0 (0.0)	75 (100.0)	75 (100.0)
RPV	3 (4.0)	1 (1.3)	9 (12.0)	4 (5.3)	10 (13.3)	6 (8.0)	53 (70.7)	64 (85.3)

## 4 Discussion

Over a 5‐year period, we reported the development of drug‐resistance mutations and a decrease in HIV susceptibility in patients who failed first‐line ARV treatment. Only two patients had five variants associated with PI resistance at the time of first‐line ART failure: one had the M46L mutation and the other had the M46I, I54V, V82A, and L10F mutations. After 5 years, we identified up to 14 HIV‐infected patients with PIs resistance mutations, with newly detected variants including L24I, I47A, F53L, G73S, T74S, I84V, N88T, L89V, and L90M. Furthermore, mutations documented in 2017 increased the number of medication resistance rates. A comparison of the level of loss in HIV sensitivity to PIs revealed a considerable decrease in sensitivity after 5 years, with LPV/r decreasing by 18.6%, ATV/r by 17.3%, and DRV/r by 6.7%. Thus, an increase of 1.3%—10.7% in the number of mutations reduces HIV sensitivity to PIs by 6.7%—18.6%.

Mutations in Gag cleavage sites may induce or contribute to PI resistance, even before protease mutations [30, 31]. As a result, a large number of HIV samples collected from patients with virological failure during PI treatment did not contain mutations consistent with PI resistance [32]. Ritonavir is now utilized at low dosages as a pharmacological enhancer for other PIs; therefore, ATV/r, DRV/r, and LPV/r are the most commonly used PIs [32]. Mutations with the highest resistance to ATV/r include I50L, I84V, and N88S, and the frequency of ATV/r‐resistant mutations increases with increasing ritonavir dosage [32]. A combination of M46I and L76V may enhance ATV/r sensitivities in the absence of other mutations [33]. In our study, I84V did now show any increase resistance to PIs, but M46I revealed very high proportion of PIs resistance.

I47V, I54M, T74P, and I84V are HIV mutations associated with resistance to DRV/r, whereas V82A enhances HIV susceptibility to DRV/r when taking boosted ritonavir [34]. These mutations have a stronger influence on resistance to PIs than other types of mutations, particularly when there are two or more mutations. V11I, V32I, L33F, I47V, I50V, I54L/M, T74P, L76V, and L89V are all linked with a lower HIV response to ritonavir‐boosted DRV/r [32]. The presence of three or more mutations, including L10F/I/R/V, K20M/N/R, L24I, L33F, M36I, I47V, G48V, I54L/T/V, V82A/C/F/S/T, and I84V, influence the HIV response to ritonavir‐boosted LPV/r. The combination of I47A/V and V32I is associated with high LPV/r resistance [33, 35, 36]. Our study also revealed that HIV samples circulating in Vietnam have mutations associated with reduced ATV/r, DRV/r, and LPV/r. sensitivity.

We found that in 2017, all 75 HIV strains had mutations that made them resistant to NRTIs, and a total of 40 mutations associated to NRTI resistance were detected, including alterations that appeared at very high rates, such as M184V (81.3%) and V75M. After 5 years, we discovered 6 new mutations, and the majority of the mutations found in 2017 increased at a faster rate. The comparison data also revealed a reduction in HIV sensitivity to NRTIs and an increase in high resistance. The meta‐analysis demonstrated that patients with the M184V mutation were 1.87 times more likely to experience virological failure. This study highlighted the unfavorable impact of the M184V mutation on treatment outcomes in PLHIV [37]. Other studies have also demonstrated that people who have HIV with M184V and V75M may have early virological failure and transmit drug‐resistant HIV strains to the wider community [25].

Mutations in the C‐terminal reverse transcriptase domain (amino acids 293–560) may lead to NRTIs and NNRTIs resistance in HIV [32]. The detection of 46 distinct mutations associated with NRTI resistance, as well as the comparison of HIV susceptibility to NRTIs, all contributed to establishing that an increase in mutations considerably lowered ART's therapeutic efficacy. Mutations such as K65E/N/R are thought to lower HIV susceptibility to TDF, ABC [38, 39], particularly when K65R is paired with M184V/I, which can reduce TDF sensitivity by less than 1.5 times [40]. T69S is resistant to all FDA‐approved NRTIs in the presence of one or more TAMs at codons 41, 210, or 215 [38]. The Q151M mutation, together with A62V, V75I, F77L, and D116Y mutations, exhibits multinucleoside resistance. TAM mutations (M41L, D67N, K70R, L210W, T215Y/F, and K219Q/E) lower HIV susceptibility to all presently licensed NRTI20s, with the exception of 3TC and FTC [41, 42]. E44D and V118I mutations can increase NRTI resistance in the presence of TAM; however, M184V is not linked with a decreased HIV response to ABC and enhances ABC resistance when paired with TAM [43, 44]. Replacement mutations T215F/I/S/Y enhance the probability of virological failure when treated with AZT in patients who have not yet started ART [45]. Therefore, HIV strains found in Vietnam have a wide range of mutations and are resistant to most of the NRTIs currently used for HIV therapy.

At the time of first‐line ART failure (2017), 32 NNRTI‐resistant mutations were detected, but in 2022 6 new mutations were detected over the next 5 years. The percentage of HIV strains resistant to NNRTIs is lower than that of NRTIs. Previous studies have shown that V106A, Y188L, and M230L reduced DOR sensitivity by more than 10‐fold [46], whereas G190E decreased DOR sensitivity by more than 20‐fold [47]. DOR resistance was significantly associated with the combination of V106A with F227L, V106A with F227L and L234I, or V106A with G190A and F227L [48]. Sixteen mutations are associated with lower HIV susceptibility to RPV (K101E/P, E138A/G/K/Q/R, V179L, Y181C/I/V, Y188L, H221Y, F227C, and M230I/L) [49, 50]. K101P and Y181I/V lower susceptibility to RPV by approximately 50‐ and 15‐fold, respectively, whereas M184I combined with E138K or K101E reduces susceptibility by about L100I paired with K103N and L100I + K103R plus V179D were highly associated with decreased susceptibility to RPV [51]. Our findings demonstrated that high rates of HIV resistance to EFV, NVP, and RPV in a 5‐year period in Vietnam, respectively. The efficacy of EFV, NVP, and RPV in treatment regimens for individuals with HIV infection resistant to NNRTIs remains unclear because of HIV's high resistance to EFV, NVP, and RPV [52, 53].

The most commonly used ART regimens for HIV therapy in Vietnam were TLD, TLE, TDF/3TC/LPV, and ABC/3TC/LPV. This study indicated that TLD and TLE were only administered when patients had virological failure, and both regimens were successful in reducing HIV replication. We also found that TLD produces more protease mutations, but patients treated with TLE did not exhibit PIs resistance mutations. Furthermore, the rate and frequency of NRTI and NNRTI resistance mutations in patients treated with LTD and LTE were nearly identical. When the pharmacological contents of the LTD and TLD regimens were compared, it was found that the TLD regimen contained DTG, which might explain why protease mutations that resist PIs used in HIV therapy exist. However, like the TLD regimen, the ABC/3TC/DTG regimen contains DTG but did not identify any HIV strains with PIs‐resistant mutations. This suggests that a single medicine may not be able to drive the development of drug‐resistant mutations in HIV, but this is dependent on a variety of conditions, including the combination of numerous ARV therapies, treatment length, treatment compliance, and the patient's constitution. Furthermore, only 1.8% of patients in this study were treated with the ABC/3TC/DTG regimen; therefore, the capacity of DTG to produce HIV protease mutations may not have been well evaluated. Therefore, assessment studies with larger sample numbers are needed to elucidate the connection between DTG and HIV protease mutations associated with PI resistance.

## 5 Conclusion

After 5 years of follow‐up, we found that HIV strains showed a considerable rise in mutations resistant to PIs, NRTIs, and NNRTIs, with the emergence of new mutations resistant to PIs outpacing the emergence of new mutations resistant to NRTIs and NNRTIs. As a result, HIV sensitivity to PIs was significantly lower than that to NRTIs and NNRTIs. This is the first study in Vietnam on monitoring and evaluating the development of drug‐resistance mutations and decreased sensitivity of HIV to antiretroviral. The results of the study will be the baseline for larger‐scale studies and provide significant contributions to knowledge, clinical practice, and HIV management and treatment alternatives.

## Author Contributions

Than Manh Hung: Data collection, formal analysis, data curation, editing, and review and validation; Le Van Nguyen Bang: formal analysis, data curation, editing, and review; Le Van Duyet: Project administration, investigation, supervision, writing – original, editing, and review and validation. All the authors have read and approved the manuscript.

## Ethics Statement

The ethics committee of the National Hospital of Tropical Diseases approved this study (approval number: 19/HĐĐĐ‐NDTW). All tests were conducted in accordance with the guidelines and regulations of the Vietnamese Ministry of Health. All participants consented to participate and signed the consent form.

## Conflicts of Interest

The authors declare no conflicts of interest.

## Supporting information


**Table S1.** Prevalence of ART resistance mutations among patients treated with different regimens.

## Data Availability

The data used to support the findings of this study are available from the corresponding author upon request.

## Appendix A

Primer set:

RT‐PCR

**Table:**

Gene	Name	Stage	Polarity	Position	Sequence	Length
PR	PR05	RT‐PCR	Forward	2074‐2095	AGACAGGYTAATTTTTTAGGGA	22
PR	PR02L	RT‐PCR	Reverse	2716‐2691	TATGGATTTTCAGGCCCAATTTTTGA	26
RT	AE_2485F	RT‐PCR	Forward	2485‐2509	GACCTACACCTGTCAACATAATTGG	25
RT	AE_3372R	RT‐PCR	Reverse	3372‐3348	TAATCCCTGCATAAATTTGACTTGC	25
INT	INT‐1st_F	RT‐PCR	Forward	4039‐4060	CAGACTCACAATATGCATTAGG	22
INT	INT‐1st_R	RT‐PCR	Reverse	5264‐5243	CCTGTATGCAGACCCCAATATG	22

Thermo cycle:

1. 45°C – 10min
2. 94°C – 2 min
3. 98°C – 10 s
4. 52°C – 20 s
5. 68°C – 26 s

30 cycles from step 3 to step 5

6 4°C – ∞

Nested‐PCR

**Table:**

Gene	Name	Stage	Polarity	Position	Sequence	Length
PR	PR01M	2nd PCR	Forward	2148‐2167	AGAGCCAACAGCCCCACCAG	20
PR	PR06	2nd PCR	Reverse	2611‐2592	ACTTTTGGGCCATCCATTCC	20
RT	AE_2574F	2nd PCR	Forward	2574‐2592	CCAGTAAMATTAAAGCCAG	19
RT	AE_3335R	2nd PCR	Reverse	3335‐3515	TCCCACTAACTTCTGTATATC	21
INT	INT‐2nd_F	2nd PCR	Forward	4146‐4148	CTGGCATGGGTACCAGCACACAA	23
INT	INT‐2nd_R	2nd PCR	Reverse	5219‐5192	CCTAGTGGGATGTGTACTTCTGAACTTA	28

Thermo cycle:

1. 94°C – 2 min
2. 98°C – 10 s
3. 54°C – 20 s
4. 68°C – 25 s
  30 cycles from step 2 to step 4
5. 4°C – ∞

Sequencing primers:

**Table:**

Gene	Name	Stage	Polarity	Position	Sequence	Length
RT	T12	Sequencing	Forward	2574‐2592	CCAGTAAAATTAAAGCCAG	19
	DRRT3	Sequencing	Forward	2931‐2951	ACTGCATTTACCATACCTAGT	21
	B2	Sequencing	Reverse	2950‐2931	CTAGGTATGGTAAATGCAGT	20
	AE_3335R	Sequencing	Reverse	3335‐3315	TCCCACTAACTTCTGTATATC	21
PR	PR01M	Sequencing	Forward	2148‐2167	AGAGCCAACAGCCCCACCAG	20
	DRPRO2L	Sequencing	Reverse	2716‐2691	TATGGATTTTCAGGCCCAATTTTTGA	26
INT	INT‐2nd_F	Sequencing	Forward	4146‐4148	CTGGCATGGGTACCAGCACACAA	23
	INT‐2nd_R	Sequencing	Reverse	5219‐5192	CCTAGTGGGATGTGTACTTCTGAACTTA	28
	IN313S	Sequencing	Forward	4542‐4557	GCAGGAAGATGGCCAG	16
	R‐4769	Sequencing	Reverse	4769‐4750	TACTGCCATTTGTACTGCTG	20

Composition of sequencing mixture:

**Table:**

Bigdye	2 μL
PCR product	1 μL
Primer (3uM)	1 μL
Distill water	6 μL
Total	10 μL

Thermal cycler:

1. 94°C – 5 min
2. 96°C – 10 s
3. 50°C – 5 s
4. 60°C – 4 min
  35 cycles from step 2 to step 4
5. 4°C – ∞
