# Supplementary material for: Development of HIV Drug‐Resistance Mutations and Antiretroviral Efficacy Among Vietnamese Patients After Failure of 5‐Year First‐Line Therapy
Source: J Clin Lab Anal. 2025 Feb 5;39(5):e25157. doi: 10.1002/jcla.25157 (PMC11904817; doi:10.1002/jcla.25157)
Supplement: Supplementary file 1 — Table S1. Prevalence of ART resistance mutations among patients treated with different regimens. [file JCLA-39-e25157-s001.docx]

**Supplementary table 1. Prevalence of ART resistance mutations among patients treated with different regimens**

|  | | **HIV mutation rate (%)/Treatment regimen** | | | | | | |
| --- | --- | --- | --- | --- | --- | --- | --- | --- |
|  |  | **ABC**  **/3TC**  **/EFV**  **(5)** | **ABC**  **/3TC**  **/LPV**  **(9)** | **ABC**  **/3TC**  **/DTG**  **(6)** | **AZT**  **/3TC**  **/LPV**  **(56)** | **TDF**  **/3TC**  **/LPV**  **(60)** | **TLD**  **(109)** | **TLE**  **(79)** |
| **INIs** |  | - | - | - | - | - | - | - |
| **PIs** | M46L/I | - | 44.4 | - | 5.4 | 15.0 | 6.4 | - |
|  | I47A/VI | - | 44.4 | - | - | 6.7 | 0.9 | - |
|  | I54V | - | 44.4 | - | 5.4 | 3.3 | 3.7 | - |
|  | L76V | - | - | - | 5.4 | - | 0.9 | - |
|  | V82A/F/T | - | 44.4 | - | 7.1 | 10.0 | 5.5 | - |
|  | I84V | - | 33.3 | - | 3.6 | - | - | - |
|  | L90M | - | - | - | - | 3.3 | - | - |
| **NRTIs** | M41L | - | - | - | 8.9 | 13.3 | 3.7 | 5.1 |
|  | E44D | - | - | - | - | 3.3 | 3.7 | 1.3 |
|  | K65R | - | 44.4 | - | 8.9 | 5.0 | 13.8 | 13.9 |
|  | D67N/E/G | - | - | - | 12.5 | 20.0 | 5.5 | 7.6 |
|  | S68G/N | - | 11.1 | - | 5.4 | 8.3 | 0.9 | - |
|  | T69DEL | - | - | - | - | 3.3 | 4.6 | 2.5 |
|  | K70N/E/G/T/R | 20.0 | - | - | 14.3 | 15.0 | 3.7 | 10.1 |
|  | L74I/V | 20.0 | - | - | 5.4 | 1.7 | 3.7 | 7.6 |
|  | V75M/I | - | 22.2 | - | 28.6 | 11.7 | 17.4 | 15.2 |
|  | F77L | - | - | - | 1.8 | - | 3.7 | - |
|  | Y115F | - | 44.4 | - | - | - | 4.6 | 5.1 |
|  | F116Y | - | - | - | - | - | 2.8 | - |
|  | Q151M | - | - | - | - | - | 2.8 | - |
|  | M184V/I | 20.0 | 66.7 | - | 32.1 | 30.0 | 19.3 | 27.9 |
|  | L210W | - | - | - | - | 1.7 | 2.8 | 2.5 |
|  | T215D/F/ILP/Y/S | - | - | - | 7.1 | 18.3 | 6.4 | 2.5 |
|  | K219E/N/Q | - | - | - | 10.7 | 13.3 | 4.6 | 5.1 |
| **NNRTIs** | A98G | 20.0 | - | - | 14.3 | 3.3 | 1.8 | - |
|  | K101P/E/Q | - | - | - | 1.8 | 10.0 | 1.8 | 3.8 |
|  | K103N/S | 20.0 | 44.4 | - | 26.8 | 25.0 | 10.1 | 20.3 |
|  | V106M/I/A | - | - | - | 7.1 | 5.0 | 9.2 | 15.2 |
|  | V108I | 20.0 | - | - | 1.8 | - | 4.6 | 10.1 |
|  | V179D/E/IT | 40.0 | - | - | 7.1 | 5.0 | 7.3 | 12.7 |
|  | Y181C/V | - | 44.4 | - | 8.9 | 20.0 | 11.9 | 15.2 |
|  | Y188L | - | - | - | - | 3.3 | 4.6 | 6.3 |
|  | G190Q/A/E | - | - | - | 16.1 | 10.0 | 5.5 | 7.6 |
|  | V196I | - | 44.4 | - | - | - | - | - |
|  | H221Y | - | - | - | 1.8 | 11.7 | 6.4 | 5.1 |
|  | P225H | 20.0 | 22.2 | - | 14.3 | 10.0 | 9.2 | 7.6 |
|  | F227FI/L | - | - | - | - | 1.7 | 0.9 | 1.3 |
|  | M230L/I | - | 22.2 | - | 5.4 | - | 0.9 | 1.3 |
|  | K238T | - | - | - | - | - | 2.8 | 3.8 |
|  | Y318F | - | - | - | 5.4 | - | 1.8 | 1.3 |
|  | N348I | - | 22.2 | - | - | 1.7 | 3.7 | 5.1 |

*INIs: integrase inhibitors; PIs: protease inhibitors; NRTIs: nucleotide revserse transcriptase inhibitors; NNRTIs: non- nucleotide revserse transcriptase inhibitors*

*TLE: (TDF/3TC/EFV); TLD: (TDF/3TC/DTG); TDF: tenofovir disoproxil fumarate; 3TC: lamivudine; LPV/r: lopinavir; AZT: zidovudine; DTG: dolutegravir; EFV:* efavirenz; *ABC: abacavir.*
